# Metagenomic analysis of the human gut virome reveals functional signatures and viral stability across hospitalized and non-hospitalized diarrheal and non-diarrheal individuals

**DOI:** 10.1186/s13099-026-00811-x

**Published:** 2026-02-22

**Authors:** Angie L. Ramírez, Luisa Páez, Laura Vega, Viviana Aya, Carolina Hernández, Nicolas Luna, Marina Muñoz, Luz Helena Patiño, Juan David Ramírez

**Affiliations:** 1 Centro de Investigaciones en Microbiología y Biotecnología-UR (CIMBIUR), School of Sciences and Engineering, Universidad del Rosario, Bogotá, Colombia; 2 Center for Global Health and Interdisciplinary Research, USF Genomics Program, Department of Global, Environmental and Genomic Health Sciences, College of Public Health, University of South Florida, Tampa, FL USA; 3 Instituto de Biotecnología, Universidad Nacional de Colombia, Bogotá, Colombia

**Keywords:** Diarrhea, Virome, Viral auxiliary metabolic genes, Hospitalization, Metagenomics

## Abstract

**Background:**

The human gut virome is a fundamental yet understudied component of the intestinal microbiome. However, its taxonomic composition and functional potential in Latin American populations remain poorly understood, particularly under clinical stressors such as hospitalization and diarrhea conditions often linked to microbial dysbiosis.

**Methods:**

We conducted a hybrid metagenomic analysis of the human gut virome from 37 fecal samples: 10 from patients admitted to intensive care units (ICU), 13 from hospitalized patients outside the ICU (Non-ICU), and 14 from non-diarrheic individuals, including taxonomic and functional profiling of viruses and detection of viral auxiliary metabolic genes (vAMGs).

**Results:**

We identified 494 high-quality viral vOTUs, from which 37,619 ORFs were predicted. Taxonomically, *Caudoviricetes* and *Intestiviridae* were consistently present across all groups, supporting their role as part of a conserved core virome. Functionally, we identified 309 putative vAMGs spanning 90 functional categories, primarily related to metabolism and environmental information processing. Non-diarrheic individuals harbored a higher number and diversity of vAMGs compared to hospitalized groups (Kruskal–Wallis, *p* < 0.01), whereas ICU and Non-ICU patients showed reduced and more variable functional profiles. Beta diversity analysis revealed that diarrhea status, rather than hospitalization per se, was associated with modest but significant shifts in functional composition (PERMANOVA, R² = 0.047, *p* = 0.025), driven by quantitative changes in shared AMGs rather than the presence of unique functions. Notably, resistance-related vAMGs, including bacitracin transporters and Zinc D-Ala-D-Ala carboxypeptidase, were detected across samples, highlighting the potential of phages as mobile reservoirs of antibiotic resistance.

**Conclusion:**

Together, our findings indicate that hospitalization and diarrhea do not markedly alter the taxonomic structure of the gut virome but are associated with modest shifts in viral functional potential. The maintenance of a stable viral community alongside variable AMG repertoires suggests that phages may modulate host–microbiome interactions primarily through functional fine-tuning rather than large-scale community restructuring. Our study provides evidence for the ecological resilience of the human gut virome and underscores the need to integrate viral communities into resistome research.

**Supplementary Information:**

The online version contains supplementary material available at 10.1186/s13099-026-00811-x.

## Introduction

The human gut virome, defined as the collection of viruses inhabiting the gastrointestinal tract, represents a highly abundant yet underexplored fraction of the microbiome, with estimates suggesting up to 10 viruses for every bacterial cell [1, 2]. Within this community, bacteriophages dominate, followed by eukaryotic viruses and dietary-derived viruses [2, 3]. Phages play an essential ecological role by modulating bacterial composition through lytic cycles or lysogenic cycles. These interactions not only shape microbial community structure but also facilitate horizontal gene transfer, through which bacteriophages can introduce new genetic material into bacterial hosts [4]. This process can confer adaptive advantages such as antibiotic resistance, increased stress tolerance, and enhanced metabolic capabilities, traits that not only promote bacterial survival but may also have significant clinical implications, particularly in the context of microbial resistance to antibiotics [5].

Despite their functional relevance, characterizing the gut virome remains challenging due to the lack of universal viral marker genes and the experimental difficulty of recovering viral particles. As a result, viral sequences, especially from Latin American populations, are underrepresented in public databases, limiting both taxonomic and functional classification [6]. Nevertheless, recent studies have proposed the existence of a “core virome” which are recurrently found across individuals regardless of health status or geographic location. This core is believed to be largely composed of temperate phages [6, 7], whose composition may shift in disease states, including inflammatory bowel diseases [8, 9], HIV infection [10], and diabetes mellitus [11].

Some researchers have even proposed that members of the core virome could be used therapeutically to restore intestinal eubiosis, the balanced state of the intestinal microbiota [12, 13, 20]. For example, Ott et al. showed that sterile fecal filtrates (FFTs) containing viral particles could reverse *Clostridioides difficile*-associated dysbiosis, likely by transferring phages that modulate bacterial communities [14] In this context, one emerging focus in virome research is the identification of vAMGs, viral genes that modulate bacterial metabolism to favor host adaptation and enhance viral replication [15]. While AMGs are well studied in marine and environmental contexts [16], their presence and role in the human gut is still incipient, and their clinical relevance remains underestimated.

Among the clinical conditions that alter the gut microbiome, hospital-acquired diarrhea is a frequent event, particularly in patients exposed to antibiotics [17] or healthcare-associated infections. This condition is often marked by reduced bacterial richness and diversity, disrupting intestinal ecosystem stability [18]. While fecal microbiota transplantation (FMT) has proven effective in restoring bacterial communities [12], therapeutic interventions remain almost exclusively bacteria-focused. In contrast, the impact and role of the virome during diarrheal episodes, especially in hospitalized patients, remain largely unexplored [19]. Given that (i) viruses are critical components of the intestinal microbiota, (ii) the virome represents a potential therapeutic target to restore balance after gut dysbiosis and (iii) little is known about its composition during diarrhea, this study aimed to characterize the gut virome using a metagenomic approach in three groups: ICU, Non-ICU, and Non-diarrheic individuals, providing insights into the structure and potential role of the virome in hospital-acquired diarrhea, an area largely understudied, particularly in Latin American populations.

## Methods

### Sample collection and inclusion criteria

A total of 23 human fecal samples were collected from patients at Hospital Universitario Mayor – Méderi in Bogotá, Colombia. All patients shared the common condition of diarrhea, defined as three or more unformed bowel movements within a 24 h [21]. Hospitalization was defined as a stay of two or more days in any of the healthcare center’s wards, as previously described [22]. The hospitalized patients were categorized into two groups: 10 samples from patients in the intensive care unit (ICU) and 13 samples from patients in other hospital wards, hereafter referred to as Non-ICU.

Additionally, 14 fecal samples were collected from apparently healthy community individuals (hereafter referred to as Non-diarrheic), who had no chronic or acute illnesses. Inclusion criteria for these participants were: (i) absence of diarrheal episodes, (ii) no use of medications, antibiotics, antiparasitics, or laxatives in the past 12 weeks, and (iii) regular bowel habits defined as 1–2 bowel movements per day without recent gastrointestinal symptoms, based on self-report. Information including age and sex of all participants enrolled in the study is provided in Supplementary Table S1.

The collection of fecal samples and associated clinical data was conducted within the framework of the project *“Descripción del Metagenoma intestinal de pacientes con diarrea atendidos en el Hospital Universitario Mayor Méderi”*, and the study was approved by the Research Ethics Committee of Universidad del Rosario (CEI-UR) under approval numbers DVO005 1856-CV1493 and DVO005 1885-CV1527.

### Nucleic acid extraction and long- and short-read sequencing

Prior to processing, fecal samples were stored at − 80 °C to preserve nucleic acid integrity. Nucleic acids were then extracted using two different methods. For Oxford Nanopore Technologies (ONT) sequencing, 250 mg of fecal material were processed with mechanical disruption beads in 200 µL of viral lysis buffer, and RNA/DNA was extracted using the Quick-DNA/RNA MagBead kit (Zymo Research, Irvine, California, USA) according to the manufacturer’s protocol. In parallel, to enable hybrid bioinformatic analysis (Illumina + ONT), DNA was also extracted using the Stool DNA Isolation Kit (Norgen Biotek Corporation, Ontario, Canada).

The quality and concentration of the extracted nucleic acids were assessed using a NanoDrop spectrophotometer by measuring the 260/280 absorbance ratio, and integrity was verified by electrophoresis on a 1.5% agarose gel.

For ONT sequencing, random hexamers were used to enhance viral sequence recovery, and libraries were loaded onto MinION flow cells (FLO-MIN114) using Kit 14 chemistry, which provides Q20+ (99%) read accuracy. Sequencing was carried out over 72 h and monitored in real time using MinKNOW V23.07.12.

DNA samples extracted with the Norgen kit were sent to Novogene (Sacramento, California, USA), where sequencing libraries were prepared using the Illumina Nextera XT DNA Library Preparation Kit and subsequently sequenced on the Illumina NovaSeq 6000 platform using 150 bp paired-end reads, yielding an average of ~ 4 Gb of data per sample.

### Bioinformatic pre-processing

For ONT-derived reads, basecalling was performed on Fast5 files using the High Accuracy setting in Guppy V.7.1.4 and the dna_r10.4.1_e8.2_400bps_sup.cfg model. Demultiplexing and low-quality read filtering (QC < 7) were also conducted in Guppy V.7.1.4. Sequencing statistics, including quality scores and average read lengths, were evaluated using NanoStat v1.6.0.

For Illumina short reads, quality checks were performed using FastQC v0.11.9 and MultiQC v1.6. Adapter trimming and low-quality read filtering were carried out with Trimmomatic v0.38 using the following parameters: ILLUMINACLIP: TruSeq3-PE.fa:2:30:10:2:TRUE, SLIDINGWINDOW:4:20, TRAILING:20, AVGQUAL:20, and MINLEN:100, running in paired-end mode with 30 threads.

To remove host-derived sequences, reads from both sequencing platforms were mapped to the human reference genome (RefSeq GCF_000001405.40) using Minimap2 v2.24 with the -ax map-ont preset, while Illumina paired-end reads were mapped using Bowtie2 v2.4.4 with the default --end-to-end and --sensitive settings. Additional mapping was performed against the SILVA_138.1 prokaryotic database to remove bacterial ribosomal RNA sequences. The filtered reads were retained for downstream analyses.

### Hybrid assembly

A hybrid assembly of Illumina and Oxford Nanopore reads was performed using SPAdes v3.15.4 in metagenomic mode (--meta) with hybrid Nanopore support (--nanopore), applying default k-mer sizes (21, 33, 55, 77) and no user-defined coverage cutoff. The overall quality of the resulting contigs was evaluated using quast v5.2.0. Viral sequences were then identified with VirSorter v2.2.4, using the parameters --include-groups dsDNAphage, ssDNA, RNA --min-length 5000 --min-score 0.5 -j 32 all. To refine these sequences and remove potential non-viral contaminants, CheckV v1.0.3 was applied, retaining only high-quality contigs (completeness > 90% and contamination < 5%).

Finally, viral contigs from each sample were clustered into viral Operational Taxonomic Units (vOTUs) using the CheckV v0.8.1 clustering workflow, which is based on all-versus-all BLASTn, pairwise ANI calculation (anicalc.py), and hierarchical clustering using aniclust.py. Clustering was performed using a threshold of 95% average nucleotide identity (ANI) and 85% alignment fraction of the shorter contig, following the official CheckV pipeline (https://bitbucket.org/berkeleylab/checkv/src/master/).

### Viral taxonomic assignment, functional annotation, and identification of viral auxiliary metabolic genes (vAMGs)

Taxonomic assignment of vOTUs at the family level was performed using PhaGCN2.3 [23]. For functional annotation, open reading frames (ORFs) were predicted using Prodigal v2.6.3 with parameters -p meta -g 11 -f gff. Predicted viral proteins were dereplicated using CD-HIT v4.7 [24] with parameters -c 0.90 -s 0.8 -n 5 -M 80,000 -g 1 -d 0 -T 32. Functional annotation was conducted against a custom database built from all viral protein records available in NCBI Virus (5,973,821 sequences). Dereplicated proteins were compared to this database using Diamond, with a minimum identity threshold of 90% and an alignment length greater than 100 amino acids. Functional annotation of vOTUs was performed using Pharokka v1.9.0 (https://github.com/gbouras13/pharokka), a phage-focused annotation pipeline, using default parameters.

Additionally, to identify potential viral auxiliary metabolic genes (vAMGs) implicated in host metabolism during viral infection, DRAM-v v1.3.5 [25] was used. To improve the reliability of the vAMG predictions, we applied an strategy proposed by An et al. 2024 [24] with minor modifications. Specifically, we retained genes with auxiliary scores between 3 and 5, while excluding those flagged as viral structural proteins, peptidases, or attachment-related genes. Additionally, we filtered out genes lacking functional annotations as well as those associated with nucleotide metabolism, nitrogen metabolism, glycosyltransferases, and ribosomal proteins, which have been previously reported as potential sources of false-positive AMGs. Functional categorization of the curated vAMGs was performed based on KEGG annotations provided by DRAM-v.

### Phylogenetic analyses

Phylogenetic analyses were conducted based on the large terminase subunit gene, one of the most commonly used viral genetic markers [26]. These analyses focused on the three most prevalent viral groups identified in the fecal samples: *Intestiviridae*, *Caudoviricetes*, and *Peduoviridae*. All available high-quality protein sequences corresponding to this gene were retrieved from the RefSeq database. When a large number of sequences were available, a random subset of 50 representative amino acid sequences per family was selected. These reference sequences were concatenated with the translated protein sequences of the vOTUs identified in this study and aligned using MUSCLE. Phylogenetic trees were inferred using IQ-TREE with maximum likelihood, applying the parameters -st AA -m TEST -bb 1000 -alrt 1000.

### Statistical analysis

Statistical analyses were performed in R v4.4.0 using the packages phyloseq, vegan, microbiome, ggplot2, and rstatix.

#### Viral community analyses

Alpha diversity of viral communities was assessed using Shannon and Simpson indices calculated from taxonomic profiles with the microbiome package. Normality was evaluated using the Shapiro–Wilk test and differences among groups were assessed using Kruskal–Wallis tests, followed by pairwise Wilcoxon rank-sum tests when appropriate.

Beta diversity was assessed using Bray–Curtis dissimilarity to assess differences in viral community composition among groups. Principal Coordinates Analysis (PCoA) was used for visualization, and statistical significance was tested using permutational multivariate analysis of variance (PERMANOVA). Differences in relative abundances at the viral family level were assessed using non-parametric Kruskal–Wallis tests.

#### Auxiliary metabolic gene (AMG) analyses

AMG profiles were constructed by counting the occurrence of distinct KEGG annotations per sample. Alpha diversity of AMGs was assessed using richness, Shannon, and Simpson indices. Normality was assessed using the Shapiro–Wilk test, and differences among treatment groups were tested using Kruskal–Wallis tests. When significant, pairwise comparisons were performed using Dunn’s test with Benjamini–Hochberg correction.

Beta diversity of AMG profiles was assessed using Bray–Curtis dissimilarity calculated from relative AMG abundances. PCoA was used for visualization and group differences were evaluated using PERMANOVA. Betadisper function was used to validate PERMANOVA assumptions and analysis of similarities (ANOSIM) was performed as a complementary test of group separation.

#### Diarrhea status analysis

To assess whether diarrhea represented a major driver of community structure, ICU and non-ICU individuals were pooled into a single diarrheic group and compared against non-diarrheic individuals. Alpha and beta diversity analyses were repeated for both viral taxonomic and AMG profiles following the same analytical framework.

All statistical tests were considered significant at *p* < 0.05.

## Results

### Study groups and recovery vOTUs from hybrid metagenomic data

In this study, we performed a comprehensive taxonomic and functional profiling of the human gut virome using a hybrid metagenomic sequencing approach. The analysis included 37 fecal samples from three groups: hospitalized patients with diarrhea admitted to intensive care units (ICU), hospitalized patients with diarrhea outside the ICU (Non-ICU), and community-dwelling individuals (Non-diarrheic).

As a result, 10,685 putative viral contigs longer than 5 kb were recovered, of which 1,421 were classified as medium- to high-quality. To reduce redundancy and approximate species-level classification, the contigs were dereplicated and clustered using a 95% sequence similarity threshold, following the criteria proposed by An et al. [24]. This process resulted in the identification of 494 high-quality viral Operational Taxonomic Units (vOTUs), defined by a completeness above 90% and contamination below 5%.

### Viral taxonomic assignment and diversity analysis

Taxonomic classification of the vOTUs was performed using PhaGCN2.3, following the ICTV 2024 classification. The analysis showed that 71.3% of the vOTUs could be assigned to at least one taxonomic level, indicating that the sequences captured a representative fraction of the viral diversity present in the human gut at the family level. However, approximately 28.7% of the vOTUs remained unclassified.

**Fig. 1:**
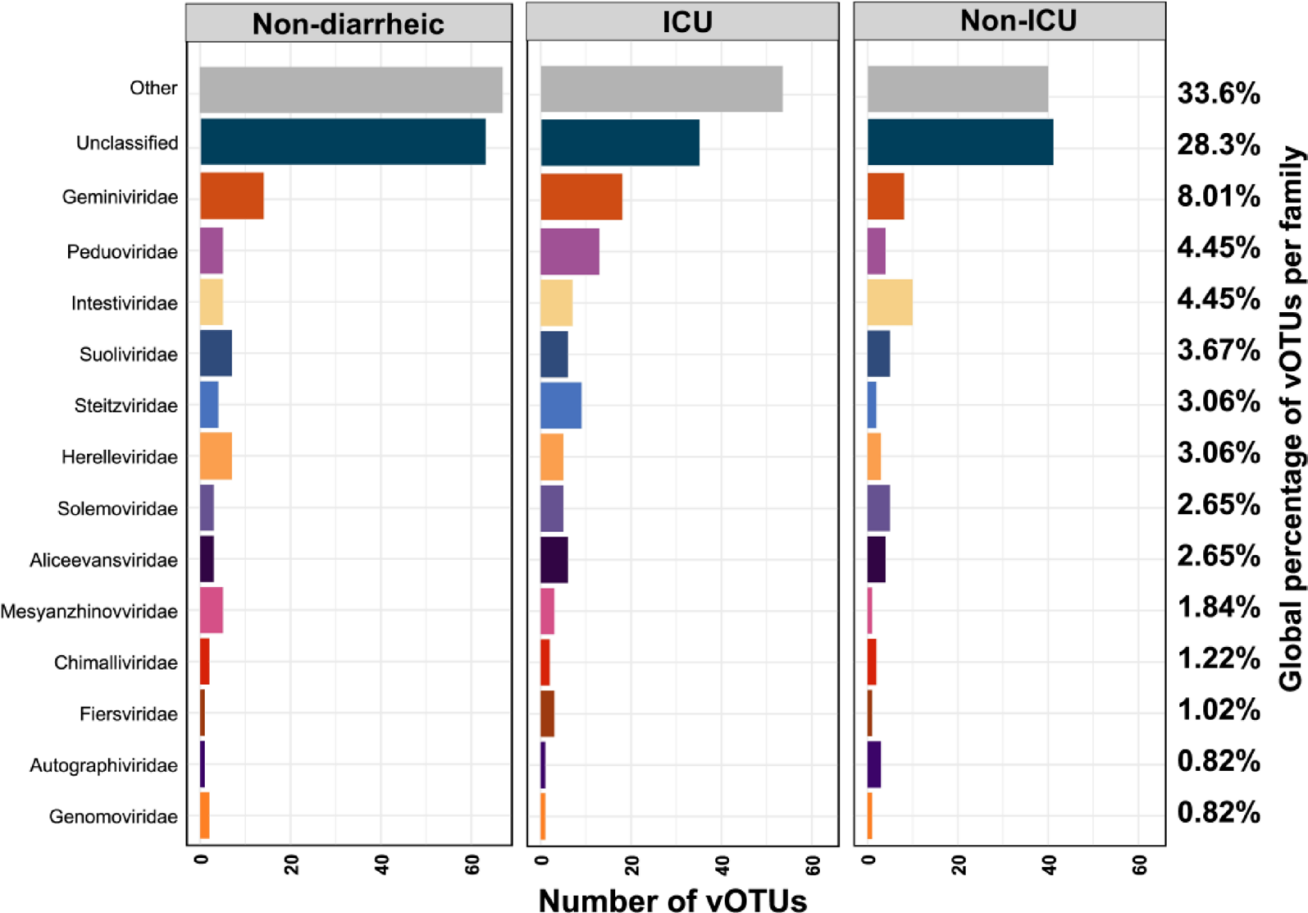
Taxonomic composition of the gut virome across study groups. Relative abundance of viral operational taxonomic units (vOTUs) classified by viral family using PhaGCN2.3 in fecal samples from Non-diarrheic (*N* = 14), Non-ICU (*N* = 13), and ICU (*N* = 10) individuals

Among the classified vOTUs, viruses belonging to the class Caudoviricetes were the most prevalent. For visualization purposes, vOTUs assigned to Caudoviricetes were grouped within the “Other” category, as they could not be resolved consistently at the family level. Other abundant viral families included Geminiviridae (8.01%), Peduoviridae (4.45%), and Intestiviridae (4.45%) (Fig. 1).

While the relative abundances of certain viral families appeared to vary across the evaluated groups, Kruskal-Wallis test revealed no significant differences between them (e.g., Caudoviricetes *p* = 0.06; Geminiviridae *p* = 0.19; Peduoviridae *p* = 0.29). At a lower taxonomic level, approximately 22% of vOTUs were classified at the genus level. Among them, the most frequently observed were *Triavirus* (*N* = 24), *Gordonvirus* (*N* = 10), *Paclarkvirus* (*N* = 8), and *Fernvirus* (*N* = 7) (Sup Fig. 1). Of these, *Triavirus* stood out, representing approximately 30% of all vOTUs assigned at the genus level. Of the 24 *Triavirus* occurrences, 13 were observed in the Non-diarrheic group, while the remaining 11 were in diarrheic subjects (6 in Non-ICU and 5 in ICU; Sup Fig. 1).

**Fig. 2:**
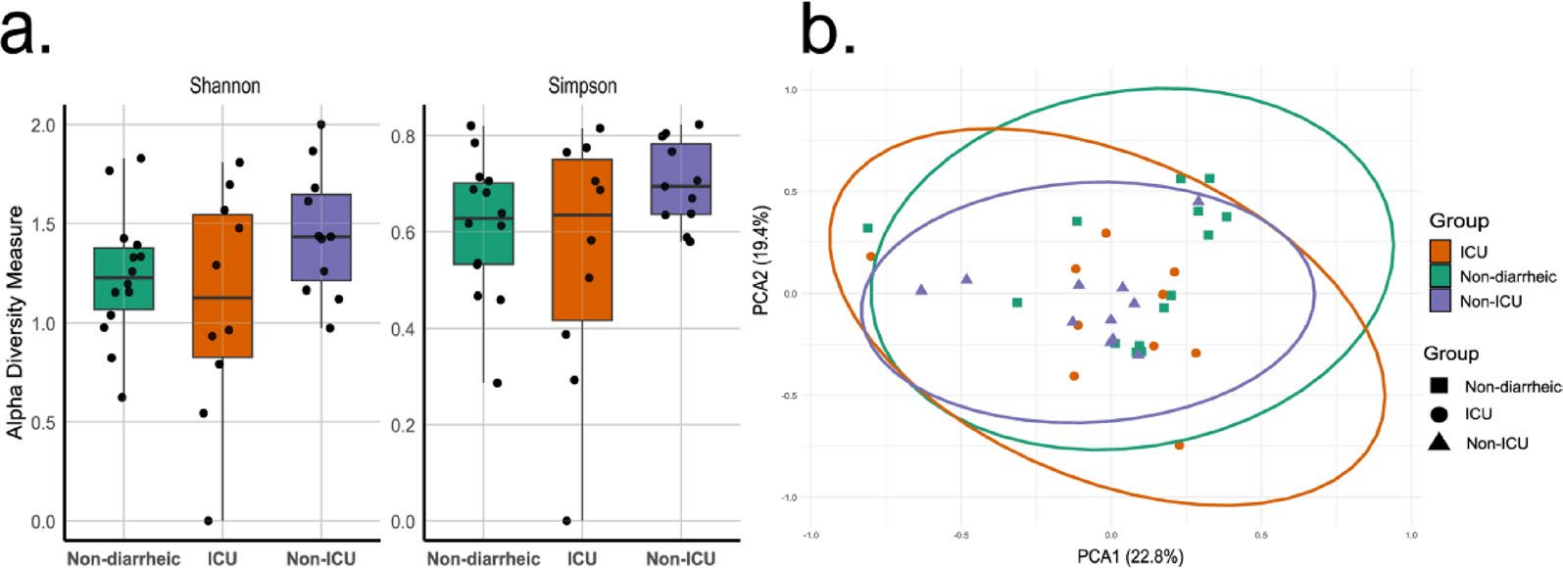
Diversity indices of the gut virome across the study groups. (**A**) Alpha diversity metrics (Shannon and Gini Simpson indices) for samples from ICU patients, Non-ICU patients, and Non-diarrheic individuals. (**B**) Beta diversity visualized through Principal Coordinates Analysis (PCoA) based on Bray-Curtis dissimilarity

Once the taxonomic profiles were established, we investigated whether the observed composition translated into differences in viral diversity across the sampling groups. Alpha diversity, assessed using Shannon and inverse–Simpson indices, was highest in the Non-ICU group (Fig. 2A); however, Kruskal–Wallis tests indicated that these differences were not statistically significant (Shannon: χ² = 3.10, *p* = 0.212; inverse–Simpson: χ² = 2.68, *p* = 0.261).

To further evaluate whether viral communities, composition varied systematically across groups, beta diversity was assessed using Bray–Curtis dissimilarity index and visualized via PCoA. No distinct clustering patterns emerged by sampling group (Fig. 2B). This was further supported by PERMANOVA, which confirmed the absence of statistically significant differences in overall community structure (*p* > 0.05). Altogether, these findings suggest that the viral communities exhibit a relatively consistent composition and distribution across the different sampling groups.

### Phylogenetic relationships of vOTUs from the most prevalent viral families

**Fig. 3:**
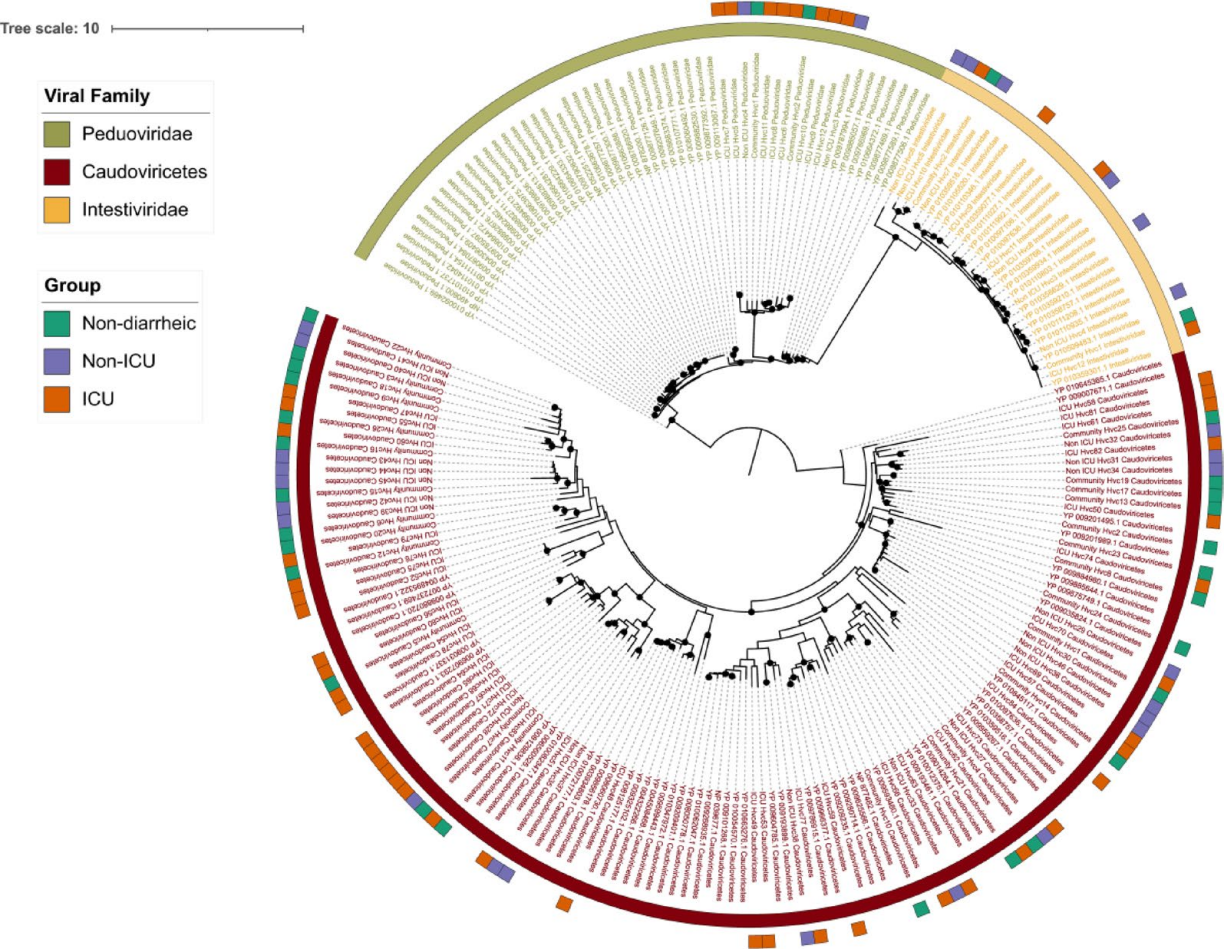
Phylogenetic reconstruction of the large terminase subunit gene from vOTUs identified in this study and reference sequences from the RefSeq database, representing the three most prevalent viral groups: *Caudoviricetes*, *Intestiviridae*, and *Peduoviridae*. Phylogenetic tree was inferred using IQ-TREE under a maximum likelihood framework. Black circles indicate nodes with SH-aLRT support values ≥ 95%

Given the absence of significant differences in viral composition and diversity among the sampling groups, we evaluated whether this pattern extended to the evolutionary structure of the most prevalent viral taxa. A phylogenetic tree was constructed using vOTUs previously classified within *Caudoviricetes*, *Peduoviridae*, and *Intestiviridae*, based on predicted open reading frames (ORFs). From these annotations, sequences corresponding to the large terminase subunit gene [26] were extracted and aligned with homologous reference sequences retrieved from NCBI. The tree was reconstructed using a maximum likelihood approach and included both study-derived vOTUs and reference sequences (Fig. 3).

The resulting topology showed that sequences consistently clustered according to their assigned viral family, forming well-supported and taxonomically coherent clades. Each of the three dominant families formed distinct phylogenetic groupings that integrated both vOTUs and reference sequences, thereby confirming the taxonomic assignments derived from PhaGCN2. Within these clades, however, vOTUs from the ICU, Non-ICU, and Non-diarrheic groups appeared interspersed, without any consistent pattern of clustering by sampling origin (Fig. 3).

### Functional profiles of the gut virome

To gain further insight into the biological roles of the viral communities, we explored their functional potential through the annotation of predicted protein-coding genes across the different sampling groups.

**Fig. 4:**
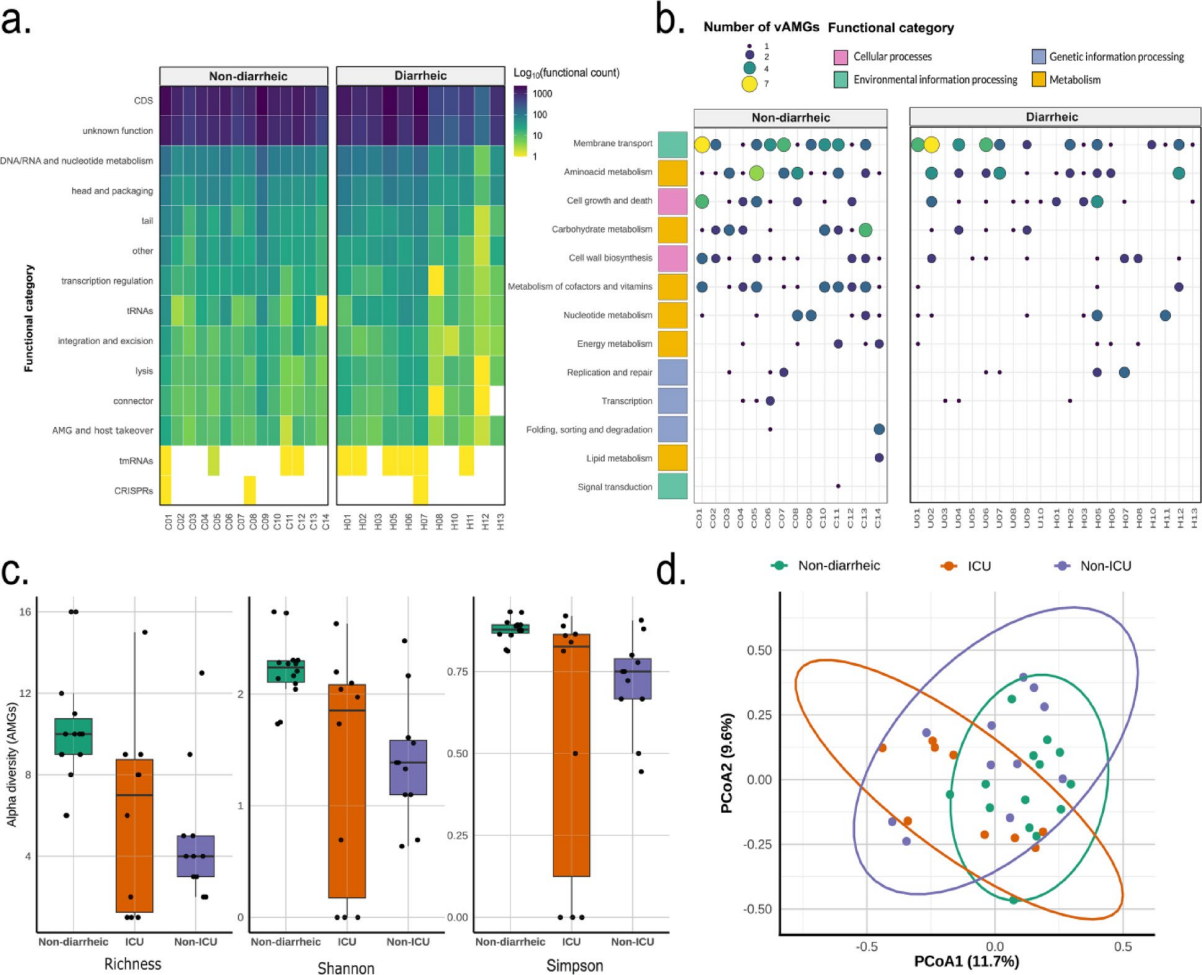
Functional Profiling of Viral Proteins and Auxiliary Metabolic Genes (vAMGs) Across Clinical Groups. **A**) Heatmap showing the distribution of viral functional categories across samples from diarrheic and non-diarrheic groups. **(B)** functional classification of vAMGs. Each row represents a specific functional category. Dot size indicates the number of vAMGs detected per function in each sample, while color indicates the broader KEGG functional category. **(C)** Alpha diversity of vAMGs across clinical groups, assessed using richness, Shannon, and Simpson indices. **(D)** Beta diversity of vAMG profiles across clinical groups based on Bray–Curtis dissimilarities

A total of 37,619 ORFs were predicted from the viral OTUs using Prodigal and clustered with CD-HIT, resulting in 34,823 non-redundant gene clusters. These vOTUs were annotated using Pharokka. Notably, 37.82% of the predicted genes were assigned to the “unknown function” category (Fig. 4A), highlighting the substantial proportion of uncharacterized genetic content in the human gut virome, despite the use of a phage-focused annotation framework.

Among the annotated fraction, most of the genes were associated with core viral functions such a DNA/RNA nucleotide metabolism, head and packaging, transcription regulation and lysis (Fig. 4A). These functional categories were detected in both diarrheic and non-diarrheic groups, with no evidence of group-specific functional enrichment.

### Detection of auxiliary metabolic genes (AMGs) in vOTUs

Beyond these essential processes, a smaller subset of annotated genes was linked to host-related functions (Fig. 4A), including those potentially involved in the modulation of host metabolism, a category that although less abundant, may be functionally relevant.

A total of 309 putative AMGs were identified across the viral gene clusters (Fig. 4B), encompassing 90 unique functional categories. Based on KEGG classification, these genes were distributed among four major functional groups: metabolism (*n* = 141), environmental information processing (*n* = 84), cellular processes (*n* = 62) and genetic information processing (*n* = 22). Among the three groups analyzed, individuals without diarrhea contributed the highest proportion of vAMG (*N* = 176), surpassing both hospitalized groups.

The most prevalent category among the AMGs corresponded to transport-related functions (*n* = 83), particularly ABC-type systems such as cell division transport system ATP-binding proteins and bacitracin transport system ATP-binding proteins, classified under environmental information processing (Suppl. Table 2). Metabolic AMGs (*n* = 79) included gene products annotated with functions related to amino acid biosynthesis, such as S-adenosylmethionine synthetase [EC:2.5.1.6] and 3-dehydroquinate synthase [EC:4.2.3.4], according to KEGG pathway assignment. Other AMGs were functionally annotated under the cellular processes category (*n* = 62), with associated pathways including cell growth and death, and cell wall biosynthesis.

Alpha diversity analysis of AMGs revealed differences among treatments for richness (Kruskal–Wallis test, *p* = 0.00201), Shannon (*p* = 0.00237), and Simpson (*p* = 0.00355) indices. Non-diarrheic samples exhibited higher AMG diversity compared to both ICU and Non-ICU samples (Dunn’s test, BH-corrected *p* < 0.0105 and *p* < 0.0041, respectively), while no significant differences were detected between ICU and Non-ICU groups (*p* > 0.69). ICU samples showed a greater spread of alpha diversity values, indicating higher within-group variability (Fig. 4C).

Beta diversity analysis based on Bray–Curtis dissimilarities revealed a significant but weak effect of Group on AMG composition (PERMANOVA, R² = 0.084, *p* = 0.017). However, ANOSIM indicated low group separation (*R* = 0.135), suggesting an overlap in AMG profiles among treatments (Fig. 4D).

Given the absence of significant differences between ICU and Non-ICU groups across alpha and beta diversity metrics, these samples were pooled into a single diarrheic group to assess diarrhea status as a primary driver of AMG diversity (Suppl. Figure 3). Under this classification, alpha diversity of AMGs was significantly reduced in diarrheic samples (Suppl. Figure 3 A) across richness (Wilcoxon rank-sum test, p_adj = 0.00088), Shannon (p_adj = 0.00088), and Simpson (p_adj = 0.00090) indices, all with large effect sizes (*r* > 0.56).

Beta diversity analysis comparing diarrheic and non-diarrheic individuals (Suppl. Figure 3B) also revealed a significant difference in AMG composition (PERMANOVA, R² = 0.047, *p* = 0.025). Nonetheless, dispersion differed between groups (betadisper, *p* < 0.001), suggesting a loss of functional consistency. Overall, these results indicate that diarrhea status is associated with reduced AMG diversity and modest compositional differences, while the core functional landscape remains largely conserved.

## Discussion

Through a hybrid sequencing approach combining Illumina short reads and Nanopore long reads, we analyzed the virome of 37 human stool samples. This strategy facilitated the recovery of extended viral contigs, improving the reconstruction of vOTUs and enabling detailed taxonomic and functional characterization of the viral communities. Additionally, it allowed the robust identification of viral functions and AMGs, providing a comprehensive view of viral communities within hospital environments.

In this study, we observed that bacteriophages constituted the most abundant viral group, regardless of hospitalization status or the presence of diarrhea (Fig. 1), a pattern consistent with previous research [27–30] reinforcing their central role as functional regulators of the microbial ecosystem. Notably, the majority of the vOTUs were assigned to Caudoviricetes (Fig. 1A), a morphologically diverse group of tailed viruses that includes both virulent and temperate phages [28]. The absence of statistically significant differences in their abundance across the three groups, suggests that Caudoviricetes represent a stable component of the intestinal virome.

This stability is particularly relevant in the clinical context of diarrhea, a frequent clinical manifestation of intestinal imbalance [18, 31], often associated with disrupted intestinal homeostasis due to antibiotic use, nosocomial infections such as those caused by *Clostridioides difficile*, or inflammatory diseases [32]. While these conditions are typically associated with reduced bacterial diversity and loss of key taxa like *Faecalibacterium*, *Roseburia*, and *Bacteroides* [33], their impact on the virome remains less explored. Our results show that, despite potential bacterial dysbiosis, certain viral groups maintain a stable presence, suggesting greater resilience.

A similar pattern was observed for the family Intestiviridae, which was consistently identified in all samples analyzed (Fig. 1A). This family has been proposed as one of the main representatives of the human core virome [34, 35]. The ubiquitous presence of Intestiviridae supports its role as a stable component of the gut ecosystem. This family includes crAssphages, prevalent viruses that infect *Bacteroides* species such as *B. intestinalis* and *B. xylanisolvens* [35, 36]. These bacteria are key to intestinal metabolism through polysaccharide degradation and short-chain fatty acid production, which may explain the persistence of their phages even during dysbiosis.

In line with this hypothesis, our phylogenetic analyses revealed that the vOTUs assigned to Intestiviridae, Caudoviricetes, and Peduoviridae consistently clustered by viral family rather than clinical condition (Fig. 3). The genetic similarity of vOTUs between hospital and community samples may reflect active horizontal transmission between populations or the long-term conservation of shared viral lineages. We hypothesize that viral communities are continually transmitted between hospitalized and non-hospitalized populations, which may help explain the absence of marked differentiation between them.

Upon further examination at the taxonomic level, just 22% of viral sequences could be assigned to the genus level. Among these, *Triavirus* stood out, representing approximately 30% of all vOTUs assigned at the genus level. Of the 24 *Triavirus* occurrences, 13 were observed in the Non-diarrheic group, while the remaining 11 were in diarrheic subjects (6 in Non-ICU and 5 in ICU; Sup Fig. 1). This pattern aligns with findings reported by Wu et. al. [37], who described a significant reduction in *Triavirus* in patients with multiple primary malignancies, including colorectal cancer, compared to healthy individuals. Although functional evidence regarding the role of this genus remains limited, these findings suggest that *Triavirus* may represent a viral component associated with an eubiotic microbiome. Future studies should explore *Triavirus* dynamics through longitudinal sampling, assessing its persistence in health and shifts during dysbiosis. Correlating its abundance with markers of eubiosis, like key taxa such as *Faecalibacterium*, could clarify its role in gut homeostasis.

To infer the functional potential of the virome, we performed gene annotation of the identified vOTUs. Most of the detected functions were associated with infection cycles, including genome replication, capsid assembly, and host infection (Fig. 4A). This functions aligns with previous findings [38] and are consistent with the Kill-the-Winner (KtW) ecological model, which suggests that phages apply selective pressure on the most abundant bacteria to prevent their overgrowth and maintain microbial balance [39].

In clinical contexts, the predominance of lytic cycles may be particularly relevant. In such scenarios, lytic viral activity could contribute to the control of opportunistic strains but may also, paradoxically, exacerbate ecological instability by destroying commensal bacteria and potentially releasing resistance genes or virulence factors [40]. The dominance of lytic-cycle functions across all clinical groups (Fig. 4A) suggests that phage-driven bacterial modulation is a core ecological process in the gut. Rather than responding solely to dysbiosis, lytic activity may help maintain microbial balance by limiting overgrowth and supporting dynamic equilibrium.

Beyond the basic functions related to viral replication, one of the most striking findings was the detection of vAMGs. A total of 309 vAMGs were identified, spanning functions related to energy metabolism, genetic information processing, cellular processes, and environmental signaling (Fig. 4B). Notably, the non-diarrheic group exhibited the highest number of vAMGs (*N* = 176), which is particularly interesting given that this same group displayed the lowest viral diversity (Shannon index; Fig. 2A).

Consistently, AMG-based alpha diversity analyses revealed significantly higher functional richness and diversity in non-diarrheic samples compared to ICU and Non-ICU groups. This indicates that increased functional potential is not necessarily linked to a more taxonomically diverse virome. In line with this, AMG composition showed substantial overlap among clinical groups, supporting the existence of a conserved viral functional core, with differences likely reflecting quantitative shifts in AMG abundance rather than major functional turnover.

Importantly, when ICU and non-ICU samples were pooled into a single diarrheic group, the same pattern was retained, with diarrheic individuals showing significantly reduced AMG diversity and increased inter-individual variability compared to non-diarrheic subjects (Suppl. Figure 3). This consistency across analytical frameworks reinforces the notion that diarrhea status, rather than hospitalization setting, represents a major driver of viral functional destabilization.

This inverse relationship between functional richness and viral taxonomic diversity suggests a more active lysogenic dynamic, possibly associated with a more stable and less disrupted microbial ecosystem. This hypothesis aligns with the ecological Piggyback-the-Winner (PtW) model [41], which proposes that in environments with high bacterial density but low viral diversity, phages tend to adopt lysogenic strategies to maximize persistence. To experimentally assess this hypothesis, phage-infected bacterial cultures could be exposed to antibiotic-induced. The prevalence of lysogeny-associated markers, such as reporter phages or integration genes, could provide evidence of whether phages adopt lysogenic strategies under unstable microbiome conditions.

Of particular interest was the detection of genes encoding ATP-dependent bacitracin transporters, which were predominantly observed in the hospitalized groups. These proteins are part of active efflux systems for toxic compounds, and their presence suggests a viral mechanism associated with antimicrobial resistance [42] in clinical settings with high selective pressure. Previous studies have shown that certain phages can carry efflux pump genes [42, 43], enabling their bacterial hosts to expel antibiotics from the cytoplasm [44]. The identification of these genes in the intestinal virome of hospitalized patients reinforces the idea that phages function not only as ecological regulators but also as vehicles for adaptive genetic resistance.

Additionally, we identified the gene encoding the enzyme Zinc D-Ala-D-Ala carboxypeptidase, which is involved in the modification of bacterial peptidoglycan by hydrolyzing terminal D-Ala-D-Ala residues. This activity prevents the binding of antibiotics such as vancomycin, thereby conferring resistance to glycopeptides [45]. Unlike efflux pump genes, Zinc D-Ala-D-Ala carboxypeptidase was detected not only in the hospitalized groups but also in the non-diarrheic group, suggesting that it may represent a broadly conserved mechanism within the intestinal virome.

Our findings highlight the need to incorporate the virome as a key component of the intestinal resistome, challenging the traditionally bacteria-centered perspective. This is particularly relevant considering the ability of viruses to act as mobile reservoirs of resistance genes [46] within the human microbiome, as evidenced by studies reporting the presence of genes associated with β-lactams, vancomycin, tetracyclines, and macrolides in viral metagenomes derived from saliva, sputum, and skin, many of which were located on phage-associated sequences [47, 48]. In the future, understanding the mechanisms that promote viral gene mobilization will be crucial for designing effective strategies for surveillance and mitigation in the face of rising antimicrobial resistance.

Despite the strengths of our hybrid sequencing approach, this study has some limitations. First, the use of a bulk metagenomic strategy likely reduced the relative representation of viral sequences, potentially limiting the detection of low-abundance viruses. The inclusion of exogenous standards to assess viral recovery efficiency would help address this limitation, particularly for low-abundance viral populations. Additionally, bacterial reads were filtered using the SILVA_138.1 database, which targets 16 S rRNA sequences; complementary filtering against complete bacterial genome databases could further improve the removal of bacterial contamination.

Second, functional annotation relied primarily on sequence-based approaches and existing reference databases, which may underestimate the functional diversity of viral genes. Emerging strategies such as protein structure prediction combined with FoldSeek-based clustering offer promising opportunities to uncover conserved viral functions that are not detectable through sequence similarity alone.

Third, experimental validation, including cloning and heterologous expression of key genes such as Zinc D-Ala-D-Ala carboxypeptidase and bacitracin transporters, would strengthen functional inferences regarding their role in antimicrobial resistance. Moreover, the inability to distinguish between community and hospital-acquired diarrhea limits clinical interpretation; incorporating this variable, along with metadata on diarrhea severity or duration, would improve the resolution of virus–host associations.

Finally, although we attempted to overcome the small sample size by pooling ICU and Non-ICU samples into a single diarrheic group, future studies with larger cohorts will be essential to more comprehensively capture fine-scale shifts in virus–host interactions in health and disease. Future studies with clinical phenotyping and longitudinal sampling will be required to directly assess the relationships between the virome and diarrhea characteristics.

## Conclusions

In summary, our findings expand current knowledge of the human gut virome by revealing its taxonomic stability across clinical conditions and its active functional role in microbial regulation. We demonstrate a high taxonomic stability dominated by Caudoviricetes, Intestiviridae and Triavirus phages, alongside a core set of viral functions involved in host infection. Notably, the non-diarrheic group exhibited a higher number of viral auxiliary metabolic genes (vAMGs), particularly those related to transport and metabolism, suggesting a more functionally diverse and stable virome in the absence of gastrointestinal symptoms. Furthermore, the widespread detection of genes associated with antimicrobial resistance highlights the potential role of viruses in the gut resistome, opening new avenues for future research into virus–host interactions in human health and disease. Together, these findings emphasize the functional relevance of the gut virome and its implications for microbial ecology and host health.

## Supplementary Information

Below is the link to the electronic supplementary material.

Supplementary Material 1

Supplementary Material 2

Supplementary Material 3: Supplementary Fig.1. Taxonomic composition of the gut Virome across study groups. Relative abundance of viral operational taxonomic units (vOTUs) classified by viral genus using PhaGCN2.3 in fecal samples from Non-diarrheic (*N* = 14), Non-ICU (*N* = 10), and ICU (*N* = 10) individuals.

Supplementary Material 4: Supplementary Fig.3. Functional profiling of viral proteins and auxiliary metabolic genes (vAMGs) according to diarrhea status. A) Functional profiling of predicted viral proteins and vAMGs comparing diarrheic and non-diarrheic individuals. **B)** Alpha diversity of vAMGs in diarrheic and non-diarrheic groups, assessed using richness, Shannon, and Simpson indices. **C)** Beta diversity of vAMG profiles between diarrheic and non-diarrheic individuals based on Bray–Curtis dissimilarities.

Supplementary Material 5

Supplementary Material 6: Supplementary Fig.2. Phylogenetic reconstruction of the large terminase subunit gene from vOTUs identified in this study and reference sequences from the RefSeq database, representing the three most prevalent viral groups: *Caudoviricetes*,* Intestiviridae*, and *Peduoviridae*. Phylogenetic tree was inferred using IQ-TREE under a maximum likelihood framework. Bootstrap support values > 95% are indicated with blue circles.

## Data Availability

The raw data from the metagenomic analysis are available in the NCBI-ENA repository under accession numbers ERA34166914 and ERA34179135.

## Abbreviations

ICU: Intensive care unit
Non-ICU: Hospitalized patients outside the ICU
FFT: Sterile fecal filtrate
FMT: Fecal microbiota transplantation
ONT: Oxford Nanopore Technologies
ORF: Open reading frame
vAMG: Viral auxiliary metabolic gene
vOTU: Viral operational taxonomic unit
PtW: Piggyback-the-Winner model
KtW: Kill-the-Winner ecological model
