# Supplementary figures and images for: Metagenomic analysis of the human gut virome reveals functional signatures and viral stability across hospitalized and non-hospitalized diarrheal and non-diarrheal individuals
Source: Gut Pathog. 2026 Feb 22;18:17. doi: 10.1186/s13099-026-00811-x (PMC12927239; doi:10.1186/s13099-026-00811-x)

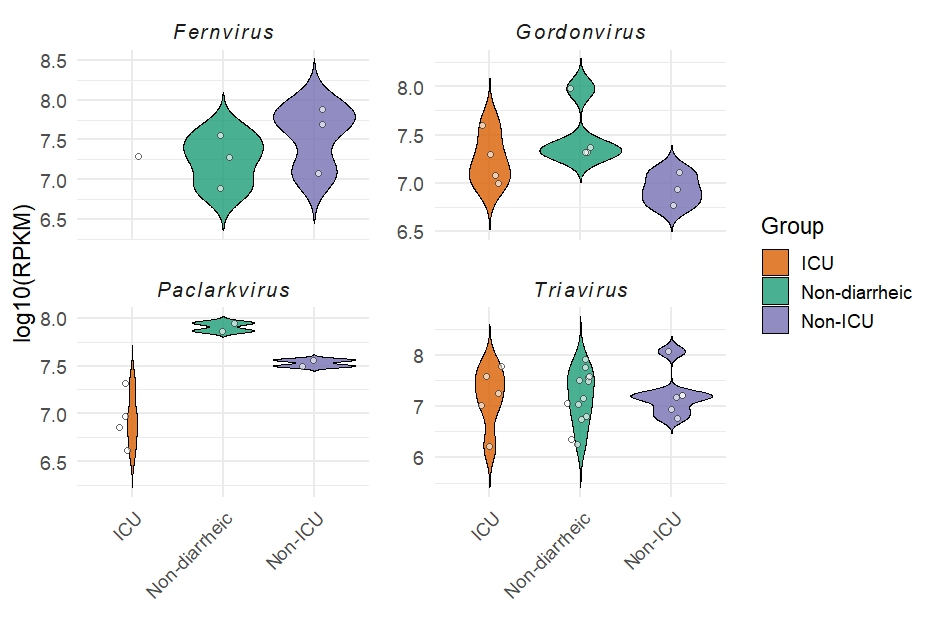

Supplement: Supplementary file 3 — Supplementary Material 3: Supplementary Fig.1. Taxonomic composition of the gut Virome across study groups. Relative abundance of viral operational taxonomic units (vOTUs) classified by viral genus using PhaGCN2.3 in fecal samples from Non-diarrheic (N = 14), Non-ICU (N = 10), and ICU (N = 10) individuals. [file 13099_2026_811_MOESM3_ESM.jpeg]
